# Physicians’ Daily Pocket Record

**Published:** 1871-12

**Authors:** 


*Physicians' Daily Pocket Record.* By S. D. Butler, M. D. Philadelphia: Office of the *Medical and Surgical Reporter.*

This is the most elegantly made and well arranged book of its kind and too much cannot be said in its favor. The manner of its closure, by spring, is a convenience worth noticing. While the tasty style of its binding and importance of its contents, make it what may truthfully be called *superior*.

